# New Insights into Evolution of Plant Heat Shock Factors (Hsfs) and Expression Analysis of Tea Genes in Response to Abiotic Stresses

**DOI:** 10.3390/plants9030311

**Published:** 2020-03-02

**Authors:** Ping Xu, Qinwei Guo, Xin Pang, Peng Zhang, Dejuan Kong, Jia Liu

**Affiliations:** 1 Department of Tea Science, Zhejiang University, Hangzhou 310058, China; zdxp@zju.edu.cn; 2 Quzhou Academy of Agricultural Sciences, Quzhou 324000, Zhejiang, China; kongxinzhu0530@163.com; 3 Suzhou Polytechnic Institute of Agriculture, Suzhou 215008, China; pxtracy916@163.com; 4 Wulanchabu Academy of Agricultural and Husbandry Sciences, Wulanchabu 012000, Inner Mongolia, China; zhangpeng9943@163.com (P.Z.); kongdejuan88@163.com (D.K.)

**Keywords:** evolutionary relationship, expression patterns, phylogenetic tree, RNA-seq, abiotic stress

## Abstract

Heat shock transcription factor (Hsf) is one of key regulators in plant abotic stress response. Although the Hsf gene family has been identified from several plant species, original and evolution relationship have been fragmented. In addition, tea, an important crop, genome sequences have been completed and function of the Hsf family genes in response to abiotic stresses was not illuminated. In this study, a total of 4208 Hsf proteins were identified within 163 plant species from green algae (*Gonium pectorale*) to angiosperm (monocots and dicots), which were distributed unevenly into each of plant species tested. The result indicated that Hsf originated during the early evolutionary history of chlorophytae algae and genome-wide genetic varies had occurred during the course of evolution in plant species. Phylogenetic classification of Hsf genes from the representative nine plant species into ten subfamilies, each of which contained members from different plant species, imply that gene duplication had occurred during the course of evolution. In addition, based on RNA-seq data, the member of the Hsfs showed different expression levels in the different organs and at the different developmental stages in tea. Expression patterns also showed clear differences among *Camellia* species, indicating that regulation of Hsf genes expression varied between organs in a species-specific manner. Furthermore, expression of most Hsfs in response to drought, cold and salt stresses, imply a possible positive regulatory role under abiotic stresses. Expression profiles of nineteen Hsf genes in response to heat stress were also analyzed by quantitative real-time RT-PCR. Several stress-responsive Hsf genes were highly regulated by heat stress treatment. In conclusion, these results lay a solid foundation for us to elucidate the evolutionary origin of plant Hsfs and Hsf functions in tea response to abiotic stresses in the future.

## 1. Introduction

In plant species, adverse environmental conditions affected seriously plant growth and development and agricultural production, such as heat, drought and cold. Among them, heat stress causes multifaceted alterations in plant germination, growth, development, physiological processes, reproduction and yield [1,2]. In plant growth, high temperature usually causes loss of cell water content, reduction in net assimilation rate (NAR) and ultimately the growth is reduced [3,4,5]. Under high temperature stress condition, plant morphological symptoms could occur, including leaf scorch, senescence and abscission, growth inhibition of shoot and root, fruit discoloration and damage [3]. However, over the long course of evolution, plants have developed numerous sophisticated mechanisms to resist this abiotic factor [6]. In recent decades, researchers reported that a lot of transcription factors in plants are participated in a mass of protective mechanisms and play a vital role in increasing the stress tolerance of crop plants [7,8,9,10,11,12]. Among them, heat shock transcription factors (Hsfs) are considered to be important regulators in the plant sensing and signaling of high temperature stress [12]. Furthermore, the studies have also shown that Hsfs are involved in plant growth and development, as well as in responses to other abiotic stresses such as cold, salt and drought [11,12,13,14,15,16].

In recent years, comprehensive identification of Hsf transcription factors from different plant species has been completed. For example, in tomato, HsfA1a plays an important role in response to high temperature [17]. Tomato HsfA2 contributes to fruit set under heat stress (HS) condition [18]. Furthermore, these two Hsfs can synergistically activate target genes by forming superactivator heterodimers [19]. In *Arabidopsis*, the expression level of HsfA9 was increased during embryogenesis and seed maturation [14], and HsfA5 can inhibit HsfA4 activity [12]. HsfA2 can not only increase the tolerance to heat stress and salt/osmotic stress [20], but also alleviate oxidative stress caused by heat stress [21] and the tolerance to anoxia [22]. In addition, HsfB1 and HsfB2b act as repressors for the expression of heat-inducible Hsfs but positively regulate the acquired thermotolerance [23]. In rice (*Oryza sativa*), HsfA4a is related to cadmium tolerance [16]; and HaHSFA9 contributes to embryogenesis in sunflowers [24]. 

Currently, despite significant variability in size and sequence of Hsfs, their structures and functions are high conserved in different plant species [25,26]. For the structure in plant Hsfs, the DNA-binding domain (DBD) and three helical bundles (α1, α2 and α3) in the N-terminus play a vital role in the positioning and recognition of heat shock elements (HSEs) [27,28,29]. The oligomerization domain (OD region), which is composed of a heptad pattern of hydrophobic amino acid residues, is connected to the conserved DBD by a flexible linker [12,30,31,32]. In addition, the nuclear localization signal (NLS), a leucine-rich nuclear export signal (NES) and a repressor domain (RD) exist in some Hsfs [12,33,34,35]. Among them, the NLS and NES were essential for nuclear import and nuclear export, respectively [12,33,34,35]. These results show the diversification of composition and function of Hsfs. However, the evolutionary relationship of Hsfs remains to be elucidated.

In the recent year, the researcher had identified Hsf gene family in different plant species, such as Arabidopsis, rice, wheat and Chinese cabbage [8,12,36,37]. However, the Hsf gene family in tea (*Camellia sinensis* L.) and their phylogenetic relationships in plant species have not been widely examined. Recently, the availability of whole genome sequencing provided an opportunity for identifying members of plant gene family [38,39,40,41]. Tea is the world’s oldest and most important caffeine-containing beverage with immense economic, medicinal and cultural importance. Recently, the genome of the tea (*Camellia sinensis* L.) has been fully sequenced [42], which provides an opportunity to further analyze the tea Hsf gene family. Therefore, in this study, we identified the comprehensive Hsf gene family members in tea and other plant species genome, inferred their evolutionary origin and explored their expression patterns in response to drought, cold and salt stresses based on RNA-seq data. Furthermore, we also performed expression patterns of Hsfs under heat stress using qRT-PCR technology, which has become the most reliable method of choice for gene expression analysis. The results will reveal the evolutionary relationship of Hsf gene family in plant species and lay a foundation for illuminating function of Hsf gene family members in tea.

## 2. Materials and Methods

### 2.1. Genome-Wide Identification of Plant Genes Encoding Hsf Proteins

Firstly, the Pfam database (http://pfam.sanger.ac.uk/) was used to locate the HSF_DNA-bind (PF00447) domains in Hsf proteins from a variety of organisms. The ClustalX 2.0 software (http://www.clustal.org/) was used to align key domain amino acid sequences, which were then used to generate the hidden Markov model (HMM) profiles for HMM searches with E-value cut-off of 1.0 against annotated protein databases from tea (http://tpia.teaplant.org/). Secondly, Hsfs in tea were identified using putative Hsfs from Arabidopsis as a query to BLAST against tea genome database. Thirdly, the HSF_DNA-bind domain in the Hsf members detected by the above two mehods (HMM or BLAST searches) was further confirmed by searching the Pfam database with E-value = 0.01 as the cut-off level. Finally, the conserved HSF_DNA-bind domain was detected using the SMART database (http://smart.embl-heidelberg.de/) with default parameters. These proteins confirmed by domain searches were regarded as putative HSF_DNA-bind domain-containing proteins; otherwise, these proteins were excluded. In addition, Hsfs from other 163 plant species were obtained transcript factor databases (http://planttfdb.cbi.pku.edu.cn/index.php), include chlorophytae (15), charophyta (1), Marchantiophyta (1), bryophyte (2), lycopodiophyta (1), coniferophyta (5), basal magnoliophyta (1), monocots (37) and eudicots (100) (Table S1). 

### 2.2. Alignment and Phylogenetic Analysis

Full-length amino acid sequences of Hsf proteins from tea and other plant species were aligned using the ClustalX 2.0 software (http://www.clustal.org/) and manually edited in BioEdit. Phylogenetic tree of the Hsf gene family was constructed using three methods (Neighbor joining-NJ, maximum likelihood-ML and Bayesian). For NJ analysis, the MEGA (version 7.0) software was used with the pairwise deletion option and Poisson correction model [43]. A bootstrap test (1000 replicates) was used to evaluate the reliability of internal branches. For ML analysis, we used the software PhyML (version 3.0) with the Whelan and Goldman amino acid substitution model, g-distribution and 100 nonparametric bootstrap replicates [44]. For Bayesian analysis, the MrBayes (version 3.2.1) software was employed to construct MrBayes trees with the fixed Whelan and Goldman model, four Markov chains and an average SD of 0.01 [45]. Additionally, for ML and Bayesian analysis, model selection was performed using the ProtTest (version 2.4) software [46].

### 2.3. RNA-Seq Data Analysis

Expression profiles of Hsf gene family members in the 16 tea plant cultivars were identified using RNA-seq data (http://tpia.teaplant.org/), including *C. assamica* cv. Yunkan 10 (CSA), *C. taliensis* (CTA), *C. tachangensis* (DC), *C. kwangsiensis* (GX), *C. crassicolumna* (HZ), *C. jingyunshanica* (JY), *C. atrothea* (LH), *C. sinensis* cv. Longjing 43 (LJ), *C. makuanica* (MG), *C. leptophylla* (MOY), *C. ptilophylla* (MY), *C. pubescens* (RC), *C. tetracocca* (SQ), *C. gymnogyna* (TF), *C. angustifolia* (XAY) and *C. parvisepala* (XE). Additionally, the public transcriptome data of apical bud, flower, fruit, young leaf, mature leaf, old leaf, root and stem have been previously generated (http://tpia.teaplant.org/). For abiotic stresses (cold, drought, MeJA, salt), expression values were measured in reads per kilobase of exon model per million mapped reads (RPKM). Then, the RPKM values were log^2^-transformed and the heat map was generated using MeV software (http://mev.tm4.org/).

### 2.4. Plant Materials and Treatments

Tea was used for all experiments in this study. The tea seedlings were planted in greenhouse and were grown under natural light and temperature conditions. The 2-year-old seedlings were used for all treatments. For the heat treatment, seedlings were subjected to 38°C conditions. Samples for RNA extractions were collected in 0, 6, 12 and 24 h time intervals. Three biological duplications were performed.

### 2.5. Expression Analysis of Tea Hsfs Using qRT-PCRs

Total RNA was extracted using QIAGEN RNeasy Mini Kit. The first-strand cDNA was synthesized using Invitrogen kit. The qRT-PCRs were carried out in 20 μL of reaction mixtures. Each reaction was composed of 2 μL cDNA, 10 μL of SYBR Green qPCR Master Mix, 0.5 μM each of primers and 7μL ddH_2_O. These reactions were carried out using Applied Biosystems. PCR reaction conditions is as follows: 94°C for 2 min, followed by 35 cycles at 94°C for 10 s, 59°C for 10 s and 72°C for 25 s. The tea *CsPTB* was used as the internal control [47]. Three technology duplications for each sample were used in this experiment, and the relative expression levels were calculated using the 2^−ΔΔ*C*T^ method [48]. All gene-specific primers used for qRT-PCR analyses were designed by Applied Biosystems Primer Express^®^ software. In addition, the standard curve method was used to calculate amplification efficiency (E) of each primer and correlation coefficient (R^2^) for all the samples tested. The equation (1 + E = 10^slope^) was used to calculate the data. All the primer sequences, E and R^2^ are listed in Table S2.

## 3. Result

### 3.1. The Tea and Other Plant Genomes Encode Different Numbers of the Hsf Family Members

To identify the tea Hsf gene family members in a genome-wide level, two methods (HMM and BLAST searches) were carried out against the whole tea genome sequences (http://tpia.teaplant.org/). The result showed that the tea genome encodes 24 HSF_DNA-bind domain-containing proteins. The details of these proteins are presented in Table 1, including locus names, genome positions, coding sequences length, amino acid sequences length, molecular weight and isoelectric point. In addition to the tea Hsf family, Hsf members from other plant genomes also identified genome-widely. The results show that the tea and other 163 plant genomes encode different numbers of the Hsf family members. A total of 4208 proteins, each containing a HSF_DNA-bind domain, in the genomes of 166 plants were identified, which were distributed unevenly into each of plant species tested. The lowest number is 1 in most of chlorophytaes green algae, and the highest number of 108 in *Camelina sativa*. All these results indicated that genome-wide variations of Hsfs had occurred during the course of evolution in plant species.

**Figure 1:**
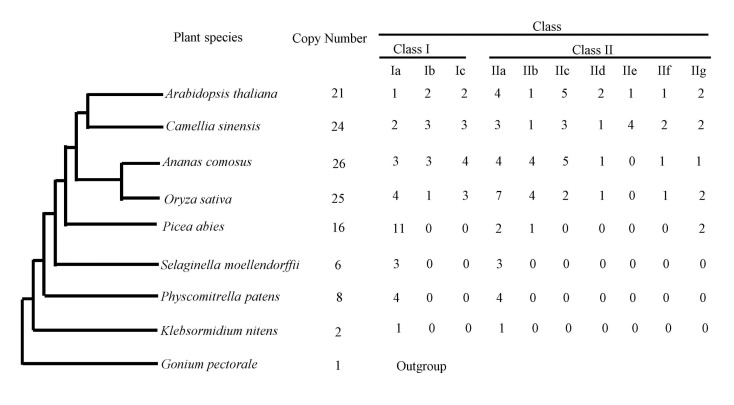
Summary of the heat shock transcription factor (Hsf) gene superfamily among 9 species.

Comparative genomic and phylogenetic analyses of Hsfs were performed in the genomes of 9 representative plant species that cover a broad diversity of plants (Figure 1). Among these lineages of plants, Hsfs are present widely in algae, bryophyta, pteridophyta, gymnosperm and angiosperms. In green algae (*Gonium pectorale*) that have been sequenced, the result indicates that it constitute a plant-specific gene family that originated during the early evolutionary history of chlorophytae algae. However, the copy number of Hsf genes varies considerably among plants, ranging from one in the green algae *Gonium pectorale* to 25 in rice (monocot) and 21 in Arabidopsis (eudicot). Therefore, rapid expansion had occurred during the course of evolution in plant species.

### 3.2. Phylogenetic Classification of Hsf Genes into Ten Subfamilies

**Figure 2:**
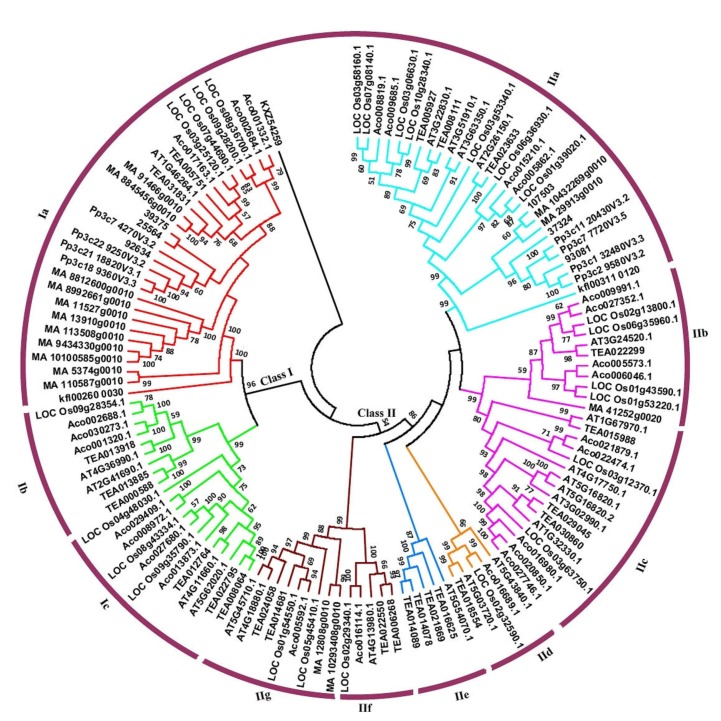
Phylogenetic tree of Hsf in plant species. Phylogenetic tree of Hsf proteins in tea plant and other plant species were generated by MEGA 7 using neighbor-joining. TEA: *Camellia sinensis*, At: *Arabidopsis thaliana*, Loc_Os: *Oryza sativa*, MA: *Picea abies*, Pp: *Physcomitrella patens*, Kf: *Klebsormidium flaccidum*, KXZ: *Gonium pectoral* and Aco: *Ananas comosus*. The protein ID of all species is listed in Table S1.

To explore the evolutionary origin of plant Hsf gene family members, phylogenetic tree with an alignment of the Hsfs from representative species were conducted using NJ, ML and Bayesian methods. Among them, ML and Bayesian analyses showed that proteins from different species cluster together in clades with high support values (not shown), with support from NJ analysis for most results. According to the results from phylogenetic, the plant Hsf genes can be divided into nine subfamilies, designated as Class Ia, Class Ib, Class Ic, Class IIa, Class IIb, Class IIc, Class IId, Class IIe, Class IIf and Class IIg (Figure 2). Among these subfamilies, Class Ia and Class IIa each contain members from the green algae to higher plants. On the other hand, Class IIb and Class IIg lack members from charophyte algae, Bryophyta and Pteridophyta. The five subfamilies, Class Ib, Class Ic, Class IIc, Class IId and Class IIf, contained members from angiosperms. In addition, Class IIe was dicot-specific group that was composed of members from *Arabidopsis thaliana* and *Camellia sinensis*.

### 3.3. Multiple Gene Duplication Events of Plant Hsfs 

To understand the evolutionary relationships of the Hsf gene family, we further analyzed the phylogeny of these Hsfs in different plant species (Figure 2). The result showed that two members (Kf1002600030 and Kf1003110120) from Class Ia and Class IIa family, respectively, were two sister groups. The chlorophytae algae, *Gonium pectorale*, as an outgroup separate from sister groups. Therefore, it is most likely that Class Ia and Class IIa were derived from gene duplication before the divergence of extant chlorophytae algaes but after the separation of chlorophytae algae from other land plants. 

Additionally, in the Class Ia subfamily, two clades with nine Hsfs (MA8812600g0010, MA8892661g0010, MA11527g0010, MA13910g0010, MA113508g0010, MA9434330g0010 and MA10100585g0010, MA5374g0010 and MA110587g0010) were gymnosperm-specific, suggesting that at least two duplication events have occurred during the course of evolution in gymnosperm. For Class Ib and Class Ic, they are from dicots and monocots. The result showed that two duplicated events had occurred in angiosperms. For Class II family, at two duplicated events had occurred in seed plants, which formed two different subfamilies (Class IIb and Class IIf) (Figure 2). Subsequently, at least one duplicated event occurred in each of these two subfamilies and formed Class IIc and Class IIg subfamilies. In addition, we also found that lineage-specific duplication in angiosperms (Class IId) and dicots (Class IIe). Together, members of Hsfs gene family in plant species experienced multiple gene duplication events during the course of evolution. 

Additionally, the difference in abundance of these Hsf members was observed among these ten classes. Class Ia is the largest one with 29 Hsfs, including 4 from eudicots (*Arabidopsis thaliana* and *Camellia sinensis*) and 6 from monocots (*Oryza sativa* and *Ananas comosus*). Class IIa was the second largest group. It was composed of 28 Hsfs including 7 from eudicots (*Arabidopsis thaliana* and *Camellia sinensis*) and 11 from monocots (*Oryza sativa* and *Ananas comosus*). In contrast, Class Ib, Ic, IIb, IIc, IId, IIe, IIf and IIg were only constituted of 9, 12, 10, 16, 5, 5, 5 and 9 genes, respectively, which was significantly less than Class Ia and IIa (Figure 2). The result showed that expansion event of plant Hsf gene family had occurred unequally in a class-dependent manner during evolution.

### 3.4. Expression Analysis of Tea Hsf Transcription Factors in Global Transcriptome 

**Figure 3:**
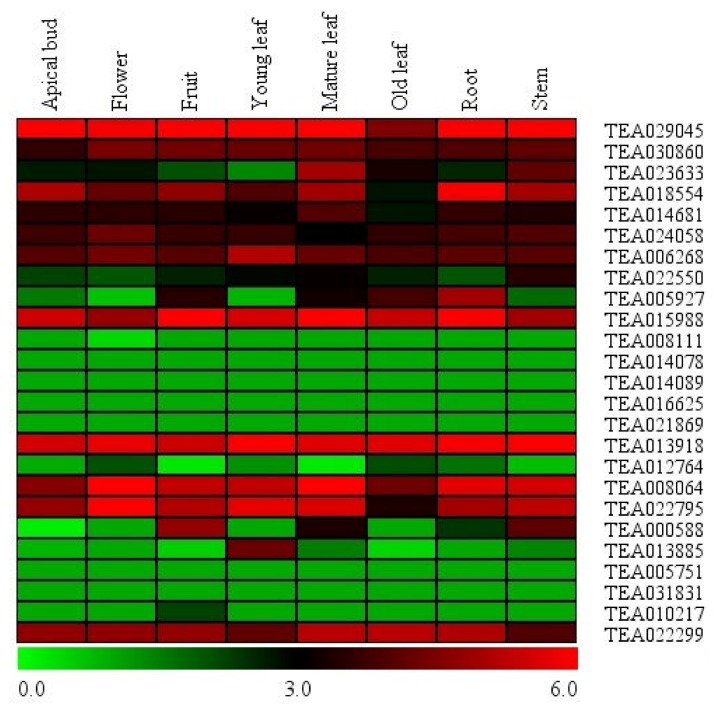
Expression patterns of Hsf genes in different tissues of tea plant. The heatmap was generated by MeV software (TIGR, Rockville, MD, USA) using the Hsf genes’ expression data, and normalized log^2^ transformed values were used with hierarchical clustering. The red and green colors indicate higher or lower transcript abundances than those of the relevant control, respectively.

To explore expression patterns of the Hsf genes in the tea tissues, a global transcription profile of the Hsf gene family members in eight organs were performed based on RNA-seq data. As illustrated in Figure 3, relative high expression levels of at least 11 Hsf gene family members were observed and the mean of the log-signal values of each gene is within the range of 3 to 6. On the contrary, nine genes show relatively low expression levels with the mean of the log-signal values of each gene ranging from zero to 1. Expression of the remaining five Hsfs showed tissue-specific. For example, expression of the TEA030860 and TEA022550 were in flower, mature leaf, old leaf and stem, TEA005927 in fruit, mature leaf, old leaf and root, TEA000588 in fruit, mature leaf and root, TEA013885 only in young leaf. These results indicated that at least half of Hsf genes were involved in plant growth and development.

### 3.5. Expression of Hsfs among Leaves of Closely Representative Camellia Species

**Figure 4:**
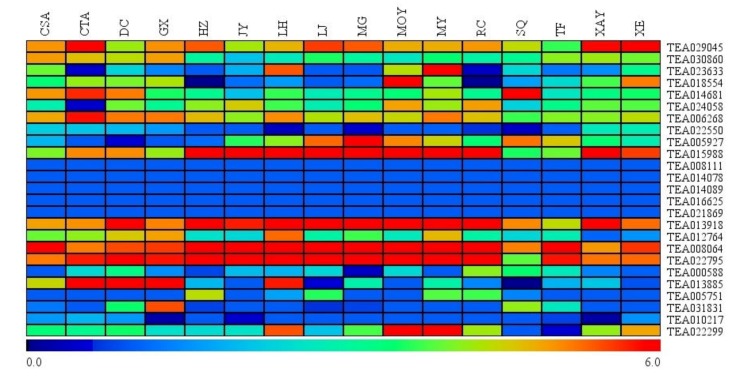
Expression patterns of Hsf genes in different *Camellia* species. The heatmap was generated by MeV software (TIGR, Rockville, MD, USA) using the Hsf genes’ expression data, and normalized log^2^ transformed values were used with hierarchical clustering. The red and green colors indicate higher or lower transcript abundances than those of the relevant control, respectively.

To further elucidate whether inter-specific expression variation of Hsf transcription factors were occurred in *Camellia*, a total of 16 closely representative *Camellia* species (*C. assamica* cv. Yunkan 10 (CSA), *C. taliensis* (CTA), *C. tachangensis* (DC), *C. kwangsiensis* (GX), *C. crassicolumna* (HZ), *C. jingyunshanica* (JY), *C. atrothea* (LH), *C. sinensis* cv. Longjing 43 (LJ), *C. makuanica* (MG), *C. leptophylla* (MOY), *C. ptilophylla* (MY), *C. pubescens* (RC), *C. tetracocca* (SQ), *C. gymnogyna* (TF), *C. angustifolia* (XAY), *C. parvisepala* (XE)) were selected for analyses. As illustrated in Figure 4, relatively high expression levels were observed in eight Hsf genes in all the samples tested, including TEA029045, TEA030860, TEA014681, TEA006268, TEA015988, TEA013918, TEA008064 and TEA022795, whereas six Hsfs (TEA008111, TEA014078, TEA014089, TEA016625, TEA021869 and TEA010217) showed low expression levels. Expression levels of the remaining 11 Hsfs vary significantly among leaves of 16 closely representative *Camellia* species. The result suggested that almost a half of Hsfs could have functional variations in different *Camellia* species.

### 3.6. Expression of Hsfs in Response to Hormone Treatment

**Figure 5:**
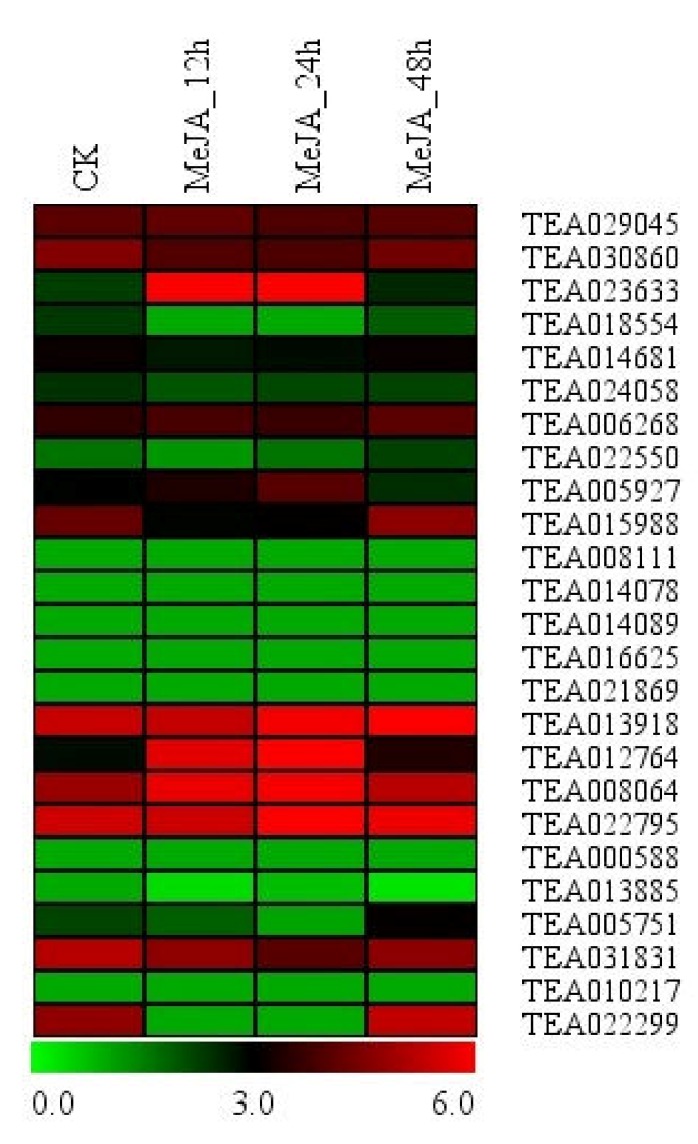
Expression patterns of Hsf genes in response to MeJA treatment. The heatmap was generated by MeV software (TIGR, Rockville, MD, USA) using the Hsf genes’ expression data, and normalized log^2^ transformed values were used with hierarchical clustering. The red and green colors indicate higher or lower transcript abundances than those of the relevant control, respectively.

Previous researches had shown that phytohormones, including auxins, cytokinins (CKs), gibberellins (GAs), salicylic acid (SA), jasmonic acid (JA), ethylene (ET), abscisic acid (ABA) and brassinosteroids (BRs), play key roles in regulating plant physiology growth, development, adaptation to environmental stimuli and intricate signaling networks involved in responses to diverse stresses [49,50,51,52,53,54,55,56]. In the current study, we investigated the expression patterns of HSF transcription factors in response to MeJA treatment. As illustrated in Figure 5, we found that expression of nineteen Hsfs was not induced by exogenous MeJA treatment. Among them, nine Hsfs showed low expression levels, while expression levels of eight were high. The remaining six genes were induced significantly by MeJA treatment. For these three Hsfs (TEA023633, TEA055927 and TEA012764), their expression levels were enhanced at MeJA_12h and MeJA_24h and decreased at MeJA_48h; while the TEA022299 and TEA015988 were the opposite. Expression levels of the TEA005751was increased at MeJA_48h. 

### 3.7. Expression of Hsfs in Response to PEG, Cold and Nacl Treatments

**Figure 6:**
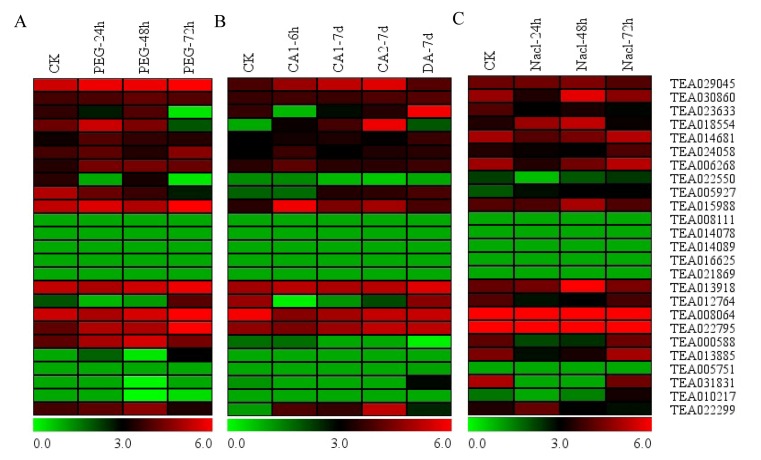
Expression patterns of Hsf genes in response to PEG (**A**), cold (**B**) and Nacl (**C**) treatments. The heatmap was generated by MeV software (TIGR, Rockville, MD, USA) using the Hsf genes’ expression data, and normalized log^2^ transformed values were used with hierarchical clustering. The red and green colors indicate higher or lower transcript abundances than those of the relevant control, respectively.

Abiotic stresses (drought, cold and salt) affected the plant growth and development and were the major factor limiting crop production [57,58,59,60,61,62,63,64]. As a kind of important transcription factor, plant Hsf proteins can regulate the expression of heat shock proteins and many other stress-related genes. The result was shown in Figure 6. To illustrate the roles of Hsf gene family members in abiotic stresses, the RNA-seq data of tea under three treatments (PEG, cold and NaCl) have been selected (Figure 6). Interestingly, similar expression patterns of most of the Hsf genes were observed in tea, whereas although some differences in expression patterns can be carefully shown. Expression levels of four Hsfs (TEA023633, TEA018554, TEA005927 and TEA02299) were increased at PEG_24h and PEG_48h and decreased at PEG_72h, while the two Hsfs (TEA012764 and TEA013855) were the opposite. Expression levels of the Hsf (TEA022550) was enhanced at PEG_48h and decreased at PEG_24h and PEG_72h (Figure 6A).

To discover the Hsf genes response to cold stress in tea, differentially expressed genes were detected using RNA-seq data. Among these Hsfs, only six genes were induced by cold treatment (Figure 6B). Expression levels of two Hsfs (TEA018554 and TEA02299) were increased at CA1-6h, CA1-7d and CA2-7d and decreased at DA-7d, while the Hsf (TEA012764) were the opposite. The expression levels of two Hsfs (TEA023633 and TEA005927) increased gradually during the cold treatment. The HSF (TEA031831) was only induced at DA-7d. 

For salt stress treatment, we found that more Hsfs were induced based on RNA-seq data (Figure 6C). The expression levels of three Hsfs (TEA012764, TEA013855 and TEA000588) decreased at Nacl_24h and Nacl_48h and increased at Nacl_72h, while the four Hsfs (TEA018554, TEA015998, TEA013918 and TEA022299) show opposite tendency. Expression levels of two Hsfs (TEA006268 and TEA005927) were increased, while the Hsf (TEA023633) was decreased gradually. In addition, two Hsfs (TEA024058 and TEA010217) were only induced at Nacl_72h. These results indicate that plant Hsf gene family members play potential roles in response to multiple stresses, including drought, cold and salt.

### 3.8. qRT-PCR Analyses of Tea Hsf Genes under Heat Stress Condition

**Figure 7:**
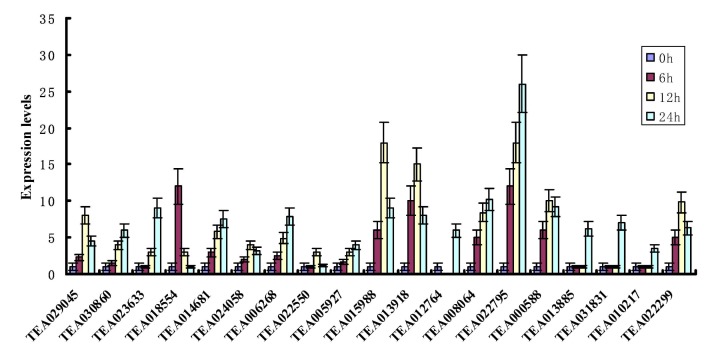
Relative expressions of tea Hsf genes under heat treatment condition. Analyses were carried out by qRT-PCR under heat stress treatment. qRT-PCR data were normalized using tea *CsPTB* [44] gene and shown relative to 0 h. X-axes showed tea Hsf genes and y-axes are scales of relative expression level (error bars indicate SD).

The plant growth and development were seriously also affected by high temperature [65,66,67,68,69,70,71,72]. In our study, nineteen of Hsf gene family members, which were expressed in different tea tissues, were selected to further confirm their expression patterns in heat stress response. The qRT-PCR was performed using tea plants exposed to high temperature (38°C) for 0, 6, 12 and 24 h. To make the data more accurate, the specificity of the primers was detected. The result showed single products of expected length using melting curve analysis method (Table S2). Moreover, in the absence of template amplification, we did not observe non-specific amplicons and no primer dimers. In addition, qRT-PCR analysis further showed that amplification efficiency (E) of the nineteen Hsf genes ranged from 0.94 for TEA018554 to 1.03 for TEA012764, correlation coefficients (R^2^) ranged from 0.9968 for TEA023633 to 0.9999 for TEA029045 and the melting temperature (Tm) ranged from 82.54 °C for TEA014681 to 86.95 °C for TEA000588 (Table S2). Therefore, these pairs of primers are suitable for detecting Hsf expression levels at high temperatures. In this study, we found that these Hsf genes expressed diversely under heat stress (Figure 7). The expression of 7 Hsfs reached its peak at 12h after heat stress, including TEA029045, TEA024058, TEA022550, TEA015988, TEA013918, TEA000588 and TEA022299; Eight genes (TEA030860, TEA023633, TEA014681, 006268, TEA005927, TEA008064, TEA022795 and TEA022795) at 24h after heat stress and only one Hsf (TEA018554) at 6h after heat stress. The remaining three Hsfs (TEA013885, TEA031831 and TEA010217) were not induced at 6 h and 12 h after heat stress treatment, but its expression level was significantly increased at 24 h.

## 4. Discussion

Plant Hsfs play key roles in the developmental regulation and in response to abiotic stresses. Although plant genomes encode multiple members, the evolutionary origin and phylogenetic relationship of Hsfs are not yet fully explored. In the current study, a comprehensive phylogenetic analysis of the Hsf gene family from algae, Bryophyta, Pteridophyta, gymnosperm and angiosperms was performed using bioinformatics method. All the genes were clustered into ten groups (Class Ia to Ic, Class IIa to IIg). Although evolution and phylogenetic relationship of Hsfs in different plant species have been reported, none of these studies addressed the possible original and the early evolution of plant Hsfs. Based on the gene number variation and phylogenetic relationship analyses of Hsf family members among different plant species, we concluded that the origin of plant Hsfs occurred in chlorophytae and a first gene duplication event occurred in the common ancestor of Charophyceae. The result indicated that this duplication event may occur in a terrestrial environment. First, one of the Hsfs from chlorophytae algae gradually evolved into the two members. Subsequently, these two members evolved into multiple members, further forming multiple subfamiles through gene expansion or genome duplication event. Moreover, we also found that these multiple gene subfamilies may occur in the ancestor of the embryophytes (Figure 2). 

To understand gene expression variation of Hsf genes in tea intra/inter-species, a total of sixteen accessions were selected for analyses. In our study, expression patterns of Hsfs showed clear differences among *Camellia* species, indicating that regulation of Hsf gene expression varied between organs in a species-specific manner. Moreover, Figure 3 also showed that, among 21 Hsfs, eight Hsfs (TEA029045, TEA030860, TEA014681, TEA006268, TEA015988, TEA013918, TEA008064 and TEA022795) have relatively high expression levels in the sixteen cultivars. Thus, we inferred that these eight Hsfs, notably for TEA008064 and TEA022795, whose RPKM value > 200 in ‘JY’, are likely participated in special physiological or biochemical pathways [23,73,74]. Additionally, the researcher also reported that, under normal/non-stress conditions, *HsfA2* with high transcript levels could activate expression of heat shock protein promoter-driven reporter genes [37]. Therefore, these two Hsfs also possibly play the indispensable roles in all the species tested since they show high expression levels (Figure 3). 

Tissue/organ-specific expression analysis can provide vital clues about the function differentiation of the genes in different tissue/organs [75,76,77,78,79,80]. In this study, to explore expression levels of Hsf family members in different tea tissues and organs, expression pattern of tea Hsfs was carried out using the previous RNA-seq data published, which was downloaded from the tea genome database (http://tpia.teaplant.org/). The result revealed that most of tea Hsf gene family members were expressed in all tissues and organs tested. Moreover, expression of some tea Hsf gene family members showed the highest level in roots and the lowest level in buds (Figure 3). All these results suggested that the Hsfs involved widely in the growth and development of tea plants. 

In the recent years, the biological functions of some Hsfs in plant response to multiple stresses have also been identified. For example, in tomato, HSFA1a, together with HSFA2 and HSFB1, regulated different aspects in the HS response and recovery. Therefore, it was considered as a master regulator for acquired thermotolerance. [18]. However, in *Arabidopsis*, none of the four HSFA1 proteins were found to play an important role as a master regulator [12]. In addition, for HSFA2 and HSFA3 in *Arabidopsis*, the research reported that these two Hsfs not only played a role in the response to heat stress, but also were involved in drought stress signaling and other stress responses [15,20]. Additionally, the qRT-PCR technology has become the most reliable method of choice for gene expression analysis in term of multiple distinct characteristics, such as a large dynamic range, high sensitivity and sequence specificity [81]. In the current study, the RNA-seq and qRT-PCR were used to investigate the expression patterns of Hsfs in abiotic stresses. The result showed that nine Hsfs (TEA029045, TEA030860, TEA014681, TEA024058, TEA006268, TEA015988, TEA013918, TEA008064 and TEA022795) enhances their expression levels under these three stress conditions including drought, cold and salt stresses (Figure 6), implying a possible positive regulatory role under abiotic stresses. Four Hsfs showed similar expression patterns under drought, cold and salt stress conditions, indicating conserved roles in response to these abiotic stresses; while seven Hsfs showed different expression patterns among these stresses, suggesting that function of tea Hsf genes were stress-specific.

Under HS conditions, plants not only accumulate different metabolites (such as antioxidants, osmoprotectants, etc.), but also activate different metabolic pathways and physiological and biochemical processes. These modifications by gene expression changes could lead to the development of heat tolerance to adaptation [82,83]. Notably, at extreme HS, due to denaturation or aggregation of the kinds of proteins, programmed cell death (PCD), which leads to the leaf shedding, or even death of the entire plant, may occur within hours or even minutes in plant species [3,14]. Therefore, in this article, we focus on, under the condition of extreme high temperature, gene expression changes of Hsfs in a short time (24h). As illustrated in Figure 7, the expression levels of the Hsf gene family members were much higher under heat treatment than under normal condition. Among them, expression level of the tea Hsf-TEA006268 was up-regulated significantly by HS, which agrees with that observed in Arabidopsis [84]. The result may explain the gradual upregulation of TEA006268 expression with prolonged HS. In addition, previous researchers reported that over-expression of HsfA6 transcription factor, which has highest expression level in all the tested Hsfs during HS, can enhance thermotolerance in wheat [37,84]. For these Hsfs from tea, we found that expression level of TEA022795 was significantly higher than that of the control and showed the largest increment among the Hsf genes tested. Therefore, we deduce that the TEA022795 gene may have a similar role in regulating the heat resistance of tea as the HsfA6 transcription factor.

## 5. Conclusions

In this study, comprehensive Hsf family members were identified in tea and other different plant species and their phylogenetic relationship from representative plant species was further investigated using bioinformatic methods. We concluded that the origin of plant Hsfs occurred in chlorophytae and a first gene duplication event occurred in the common ancestor of Charophyceae. In addition, the expression profiles of Hsfs suggested that majority Hsfs were involved in development of underground parts and regulation of reproductive growth in tea. Furthermore, significant divergence of expression levels of Hsf genes was observed under different abiotic stress conditions. These identification and function analysis will serve a better understanding of molecular basis for tea genetic improvement and evolution relationship in plant species.

## Figures and Tables

**Table 1:** Key features of heat transcription factors (HSFs) in *Camellia sinensis*.

Gene Name	Locus	Scaffold	ORF Length ^a^ (bp)	Deduced Polypeptide		
				Length ^b^ (aa)	Mol wt ^c^ (kDa)	*pI* ^d^
CsHSF01	TEA000588	Scaffold4738:242479-245432	723	240	27.83	5.59
CsHSF02	TEA005751	Scaffold3668:1248898-1251806	849	282	32.80	6.42
CsHSF03	TEA005927	Scaffold372:5052384-5054777	1059	352	40.55	5.37
CsHSF04	TEA006268	Scaffold1096:351535-356817	1440	479	53.66	5.76
CsHSF05	TEA008064	Scaffold385:66932-69032	900	299	34.02	6.27
CsHSF06	TEA008111	Scaffold736:2759744-2761297	1023	340	38.81	5.07
CsHSF07	TEA010217	Scaffold4444:1140231-1146979	519	172	19.44	10.01
CsHSF08	TEA012764	Scaffold474:257888-260307	945	314	35.08	5.37
CsHSF09	TEA013885	Scaffold598:1002357-1004559	723	240	27.87	6.67
CsHSF10	TEA013918	Scaffold598:1726479-1739146	1569	522	57.83	5.16
CsHSF11	TEA014078	Scaffold2655:1213850-1216317	996	331	37.20	5.14
CsHSF12	TEA014089	Scaffold2655:1252903-1263984	1401	466	51.34	4.49
CsHSF13	TEA014681	Scaffold2339:167132-178145	1998	665	74.98	5.91
CsHSF14	TEA015988	Scaffold2960:495401-502597	1167	388	44.26	4.72
CsHSF15	TEA016625	Scaffold2245:1443845-1445616	1116	371	41.43	4.71
CsHSF16	TEA018554	Scaffold367:1317907-1320377	1722	573	63.81	5.02
CsHSF17	TEA021869	Scaffold151:757381-759282	1266	421	47.11	5.16
CsHSF18	TEA022299	Scaffold881:562868-564244	966	321	36.25	5.35
CsHSF19	TEA022550	Scaffold502:2406177-2409890	684	207	23.56	5.68
CsHSF20	TEA022795	Scaffold3997:691331-693719	1104	367	40.70	5.35
CsHSF21	TEA023633	Scaffold1243:1037225-1040235	1203	400	45.39	4.85
CsHSF22	TEA024058	Scaffold1471:1329221-1337467	1470	489	55.56	5.00
CsHSF23	TEA029045	Scaffold1047:271898-276789	1587	508	56.46	5.13
CsHSF24	TEA030860	Scaffold2384:70221-80010	1542	513	56.78	4.78
CsHSF25	TEA031831	Scaffold2436:227948-229420	906	301	33.38	6.49

^a^ Length of open reading frame ^b^ Length (no. of amino acid) of the deduced polypeptide ^c^ Molecular weight of the deduced polypeptide in Dalton ^d^ Isoelectric point of the deduced polypeptide.

## Supplementary Materials

The following are available online at https://www.mdpi.com/2223-7747/9/3/311/s1. Table S1: Amino acid sequences of Hsf proteins in all the genomes tested. Table S2: Primers used to detect the expression of tea Hsf genes in this study.
