# Supplementary material for: New Insights into Evolution of Plant Heat Shock Factors (Hsfs) and Expression Analysis of Tea Genes in Response to Abiotic Stresses
Source: Plants (Basel). 2020 Mar 2;9(3):311. doi: 10.3390/plants9030311 (PMC7154843; doi:10.3390/plants9030311)
Supplement: Supplementary file 1 [file plants-09-00311-s001.zip › Supplemental Table S2.doc]

Table S2: Primers used to detect the expression of tea Hsf genes in this study.

| Name | Forward primer (5’ to 3’) | Reverse primer (5’ to 3’) | Amplicon length (bp) | E | R^2^ | Tm (°C) |
| --- | --- | --- | --- | --- | --- | --- |
| TEA029045 | GCACCGTTGAAGTCTCTGAC | TTATGCCTGGAAGCTTGGGA | 165 | 0.99 | 0.9999 | 83.04 |
| TEA030860 | ACGACTCCCTTTCCAACCAT | GAGAGAGCTCGGGCATAACT | 76 | 0.97 | 0.9986 | 84.65 |
| TEA023633 | CCGTTTCTCAGCAAGACGTT | TGAGAGTCCCAAACGACGAA | 101 | 0.99 | 0.9968 | 85.12 |
| TEA018554 | CCGGAGTTCTTAGCTCGTCT | GGTTCCACAACTCGGGTCTA | 160 | 0.94 | 0.9989 | 83.16 |
| TEA014681 | CGTGGAGTCCGTCAAACTTC | CAGAAGACACAGCCAACTCG | 199 | 0.97 | 0.9994 | 82.54 |
| TEA024058 | GTCCTCTTGCTCCTGAGGTT | CCCAGAACACATCGTTGACC | 90 | 0.98 | 0.9998 | 84.65 |
| TEA006268 | TTACTCCCAGCCAGTTCCAG | AGTCTCCTTTGAGGGCTTCC | 195 | 0.96 | 0.9978 | 86.35 |
| TEA022550 | AACAACAGCATCGTGGTCTG | GCTGGAGAAGTTGTTGTGCT | 84 | 1.01 | 0.9992 | 83.25 |
| TEA005927 | CAAGGGACAGAGAGGAAGCA | CTTCGACTGGATTGGCCTTG | 173 | 0.98 | 0.9995 | 84.31 |
| TEA015988 | CACTGGGAATTTGCGAACGA | TGCTCCAACACTCTTGTCCT | 132 | 0.95 | 0.9989 | 85.64 |
| TEA013918 | GGCACTGGGCTTTGAAATGA | TGGTCAATGATGTGGAACGC | 171 | 0.99 | 0.9994 | 84.95 |
| TEA012764 | TTCGTTCGACAGCTCAACAC | AGCACTTTCTCGCCTCTCTT | 95 | 1.03 | 0.9986 | 86.58 |
| TEA008064 | GCCCGAGATTGTTTGGAGTC | ATGATGCGCCTCACTTTCAC | 71 | 0.97 | 0.9988 | 86.38 |
| TEA022795 | AGCAGAACGGTGGAGAATCA | CGGATTCCAGACGATGAACG | 152 | 0.99 | 0.9987 | 84.61 |
| TEA000588 | GTCCTCACCACCACCTTTCT | CTCGGAGTTCCACGAGATGA | 85 | 0.95 | 0.9996 | 86.95 |
| TEA013885 | GATATCGTGGAACGCCGATG | AGCTGCCTGACAAAGCTAGA | 117 | 0.98 | 0.9992 | 84.69 |
| TEA031831 | TTGCCCACATGAGGAAGCTA | GGAGTAGCACAGGATGGTGA | 121 | 0.96 | 0.9993 | 86.49 |
| TEA010217 | GTGGTGATGGTAGTGGTGGA | ATGTGCTAGGCAACATGAGC | 111 | 1.02 | 0.9997 | 86.05 |
| TEA022299 | GGACCGTTTCATCAGTTGGG | GCGAACGAAGCTGGAGAAAT | 121 | 0.99 | 0.9989 | 85.95 |

E, amplification efficiency; R^2^ , correlation coefficient; Tm, melting temperature.
